# A Novel Inflammation-Based Prognostic Score, the C-Reactive Protein/Albumin Ratio Predicts the Prognosis of Patients with Operable Esophageal Squamous Cell Carcinoma

**DOI:** 10.1371/journal.pone.0138657

**Published:** 2015-09-21

**Authors:** Xiao-Ling Xu, Hui-Qin Yu, Wei Hu, Qian Song, Wei-Min Mao

**Affiliations:** 1 Department of Medical Oncology, Zhejiang Cancer Hospital, 38 Guangji Road, Hangzhou City, China; 2 Digestive Department, Hanggan Hospital, Hangzhou City, China; 3 Department of Respiration, The Second of Yuhang People’s Hospital, Hangzhou City, China; 4 Department of Clinical Laboratory, Zhejiang Cancer Hospital, 38 Guangji Road, Hangzhou City, China; Zhongshan Hospital Fudan University, CHINA

## Abstract

**Background:**

Inflammation-based prognostic scores such as the neutrophil lymphocyte ratio (NLR), platelet lymphocyte ratio (PLR), Glasgow prognostic score (GPS), and modified GPS (mGPS) have been reported to have prognostic value in patients with many types of cancer, including esophageal squamous cell carcinoma (ESCC). However, the role of the C-reactive protein/Albumin (CRP/Alb) ratio in ESCC has not yet been evaluated.

**Methods:**

A total of 468 patients suffering from histologically proven ESCC were enrolled between January 2000 and July 2010. The GPS, mGPS, NLR, PLR and CRP/Alb ratios were tested together with established prognostic factors in univariate and multivariate Cox regression analyses of overall survival (OS).

**Results:**

The optimal cutoff level for the CRP/Alb ratio was 0.50. The CRP/Alb ratio (continuous) had higher AUC values at 12 months (0.796), 24 months (0.805), and 36 months (0.815) than the NLR, GPS and mGPS. In univariate analysis, the 5-year OS rate for patients with a CRP/Alb ratio > 0.50 was 43.4%, while the rate for patients with a CRP/Alb ratio ≤ 0.50 was 17.7% (*P* < 0.0001). In multivariate analysis, patients with a CRP/Alb ratio > 0.50 had worse survival than patients with a CRP/Alb ratio ≤ 0.50 (HR: 2.44; 95% CI: 1.82–3.26; *P* < 0.0001).

**Conclusion:**

In summary, to the best of our knowledge, this is the first study to identify the CRP/Alb ratio as a novel inflammation-based prognostic factor in a large group of ESCC patients. The prognostic value of the CRP/Alb ratio needs to be verified in prospective multicenter studies.

## Introduction

Esophageal cancer (EC) is the eighth most prevalent malignancy in the world with an incidence that continues to rise [1]. EC is one of the leading causes of cancer-related mortality worldwide, causing over 406,800 deaths per year. The major pathologic subtype of EC in China is esophageal squamous cell carcinoma (ESCC). With improvements in early detection and surgical technologies, surgery has become the most effective therapy for ESCC [2, 3]. However, ESCC is still associated with a poor prognosis [2, 3]. Many biomarkers [4–6] that have been evaluated using various methods such as immunohistochemistry have been shown to better predict prognosis. However, because of conflicting results that have emerged from independent studies, the reliability of these prognostic indicators in ESCC remains controversial. New biomarkers that can complement and improve upon current strategies for ESCC detection are urgently needed.

Growing evidence indicates that inflammation plays an important role in tumorigenesis. An inflammatory microenvironment is an essential component of tumors [7]. Cancer-related inflammation can influence cell proliferation, tumor cell migration, invasion, metastasis, cell survival, angiogenesis, etc. [8]. Elevated levels of C-reactive protein (CRP), which is a marker of systemic inflammation, was found to be a predictor of low survival in patients with various cancers, including ESCC [9–11]. In the last few years, inflammation-based prognostic scores, including the neutrophil lymphocyte ratio (NLR), platelet lymphocyte ratio (PLR), Glasgow prognostic score (GPS), and modified GPS (mGPS), have been reported to have prognostic value in many cancers, including EC [9, 12–14].

Recently, the CRP/Albumin (CRP/Alb) ratio was reported to correlate with poor prognosis in patients with hepatocellular carcinoma [15]. However, the role of the CRP/Alb ratio has not yet been evaluated in surgically resected ESCC patients. In the present study, we have evaluated and compared the prognostic value of a panel of inflammatory biomarkers that include the NLR, PLR, GPS and mGPS in a Chinese population with resectable ESCC. In addition, we compared the novel prognostic factor, the CRP/Alb ratio, with other established inflammation-based prognostic indices.

## Materials and Methods

### Patients

Written informed consent was obtained from all patients enrolled in this study. The study was approved by the Ethics and Scientific Committees of Zhejiang Province Cancer Hospital. Between January 2000 and July 2010, 468 patients suffering from histologically proven EC were enrolled in this retrospective study in Zhejiang Cancer Hospital. Blood samples were obtained before surgery to measure CRP and albumin levels as well as the white blood cell count, neutrophil count, lymphocyte count, and platelet count. The following eligibility criteria were used: (1) surgery included radical esophagectomy; (2) patients survived at least 30 days postoperatively; (3) the primary tumor was located in the thoracic esophagus; (4) no other cancers had arisen in other organs; and (5) patients did not receive any neoadjuvant therapy. Patients who had any form of acute infection or chronic inflammatory disease (e.g., vasculitis, connective tissue disorders, or rheumatological conditions) were excluded. The patients who had risk factors after surgery received further adjuvant radiotherapy or chemotherapy. The following clinicopathological factors were selected and evaluated: age, gender, smoking, alcohol consumption, venous/lymphatic invasion, perineural invasion, adjuvant radiotherapy or chemotherapy, tumor size, TNM stage (American Joint Committee on Cancer 7th edition [16]) and tumor differentiation.

Use of the GPS was proposed by previous studies [17–19]. Briefly, patients with elevated C-reactive protein (> 10 mg/l) and hypoalbuminemia (< 35 g/l) were given a score of 2. Patients who had abnormal values of only one of these biological indicators were given a score of 1. Patients who had no abnormalities in either of these biological indicators were given a score of 0. The mGPS, which was reported in a previous study [20], was calculated using the CRP and albumin values as follows. Patients with elevated C-reactive protein (> 10 mg/l) were given a score of 1 or 2 depending on the absence or presence of hypoalbuminemia (< 35 g/l). Patients with a normal CRP and any albumin level were given a score of 0 [20]. The NLR was defined as the neutrophil count divided by the lymphocyte count, and the PLR was calculated by dividing the platelet count by the lymphocyte count [21]. The CRP/Alb ratio was calculated by dividing the serum CRP level by the serum albumin level [22].

CRP levels were measured using a latex particle enhanced immunoturbidimetric assay (Sekisui Chemical, Osaka, Japan) according to the manufacturer’s instructions. A serum CRP concentration of more than 5 mg/l was considered pathological. Albumin levels were measured using the bromocresol green (BCG) assay (Sekisui Chemical, Osaka, Japan). Serum albumin concentrations of lower than 35 g/l were considered pathological.

Comparisons between groups were performed using the Chi square test. The optimal cutoff level for CRP/Alb ratio was determined by receiver operating characteristic (ROC) analysis. The areas under the curve (AUC) were calculated and compared using the method reported by DeLong *et al*. [23] The overall survival (OS) was measured from the date of surgery to death or last living contact. Patient survival data were obtained from hospital records and telephone interviews with the patients. Univariate survival analysis was performed using the log-rank test, and Kaplan-Meier estimates were shown in the resulting figures. We used the Cox proportional hazards model for multivariate analyses and backward stepwise selection in Cox modeling. AUC analyses were performed using MedCalc® statistical software version 15.2.1 (MedCalc Software bvba, Ostend, Belgium). Other analyses were performed using SPSS software version 18.0 (SPSS Inc., Chicago, IL, USA). All of the tests were two-sided, and *P* < 0.05 was considered statistically significant.

## Results

### Patient characteristics

A total of 468 subjects were selected (most of whom were male), with a median age of 58 years. All patients had a Karnofsky performance status of ≥ 90 and were physically suited for surgery. Tumor locations were mainly in the middle (43.6%) and lower (53.2%) esophagus. Of the total number of cases, 51 (10.9%) were classified as well-differentiated, 3321 (70.9%) as moderately differentiated, 85 (18.2%) as poorly differentiated and undifferentiated tumors. The AJCC lymph node stage was N1 in 31%, N2 in 24.8%, and N3 in 7.7% of patients. According to clinical criteria, a total of 196 patients received adjuvant radiotherapy or systemic chemotherapy. Combined adjuvant chemoradiotherapy was initiated in 56 subjects.

The GPS score was 0 in 336 cases (71.8%), 1 in 101 cases (21.6%) and 2 in 31 cases (18.5%) **(Table 1)**. On the basis of preoperative complete blood testing, 89 patients (19.0%) were hypoalbuminemic (median 37.8 g/ l, range 20.1–53.0 g/l) while 108 (23.0%) patients had a CRP >10 mg/l (median 6 mg/l, range 0.1–241.0 mg/l). According to the mGPS, 31 (18.5%) patients had both hypoalbuminemia and high CRP levels (mGPS = 2), 77 (16.4%) patients had high CRP levels but normal albumin (mGPS = 1), and the remaining 360 (76.9%) patients had neither risk factor (mGPS = 0). The association between CRP/Alb ratio and the characteristics of patients with resectable ESCC is shown in **Table 1.** The results indicated that a higher CRP/Alb ratio was associated with male gender (*P* = 0.032), more lymph node metastasis (*P* < 0.0001), more advanced clinical stage (*P* < 0.0001) and venous/lymphatic invasion (*P* = 0.013) (**Table 1**). In addition, CRP/Alb ratio was associated with other inflammatory biomarkers, including GPS, mGPS and NLR (all *P* value < 0.0001) but not PLR (*P* value < 0.064).

**Table 1:** Correlation of the CRP/Alb ratio with the clinicopathological characteristics of ESCC patients.

Characteristics	Number of patients	No. of Patients (%)		*P* value
		CRP/Alb ratio < 0.50	CRP/Alb ratio > 0.50	
**Gender**				0.032
**Male**	416	333(87.4)	83(95.4)	
**Female**	52	48(12.6)	4(4.6)	
**Age**				0.962
**< 58 years old**	227	185(48.6)	42(48.3)	
**≥58 years old**	241	196(51.4)	45(51.7)	
**Smoking**				0.231
**Never**	109	93(24.4)	16(18.4)	
**Ever**	359	288(75.6)	71(81.6)	
**Alcohol consumption**				0.463
**Never**	150	125(32.8)	25(28.7)	
**Ever**	318	256(67.2)	62(71.3)	
**Differentiation**				0.086
**Well**	51	43(11.3)	8(9.2)	
**Intermediate**	331	275(72.3)	56(64.4)	
**Poor or undifferentiated**	85	62(16.3)	23(26.4)	
**Tumor size**				0.549
**<5 cm**	338	278(73.0)	60(69.8)	
**≥5 cm**	129	103(27.0)	26(30.2)	
**T stage**				0.145
**T1**	20	17(4.6)	3(3.5)	
**T2**	73	65(17.7)	8(9.4)	
**T3**	360	286(77.7)	74(87.0)	
**Lymph node metastasis**				<0.0001
**N0**	171	153(40.1)	18(20.7)	
**N1**	145	124(32.5)	21(24.1)	
**N2**	116	84(22.0)	32(36.8)	
**N3**	36	20(5.2)	16(18.4)	
**Clinical stage**				<0.0001
**I**	24	20(5.2)	4(4.6)	
**II**	181	164(43.0)	17(19.5)	
**IIIA**	121	100(26.2)	21(24.1)	
**IIIB+IIIC**	142	97(25.5)	45(51.7)	
**Venous/lymphatic invasion**				0.013
**No**	355	298(78.2)	57(65.5)	
**Yes**	113	83(21.8)	30(34.5)	
**Perineural invasion**				0.132
**No**	317	264(69.2)	53(60.9)	
**Yes**	151	117(30.7)	34(39.0)	
**Adjuvant radiotherapy or chemotherapy**				0.391
**No**	272	225(59.1)	47(54.0)	
**Yes**	196	156(40.9)	40(46.0)	
**GPS**				<0.0001
**0**	336	310(81.4)	26(29.9)	
**1**	101	64(16.8)	37(42.5)	
**2**	31	7(1.8)	24(27.6)	
**mGPS**				<0.0001
**0**	360	331(86.8)	29(33.3)	
**1**	77	43(11.3)	34(39.1)	
**2**	31	7(1.8)	24(27.6)	
**NLR**				<0.0001
**≤2.40**	204	191(50.1)	13(14.9)	
**>2.40**	264	190(49.9)	74(85.0)	
**PLR**				
**≤147**	283	238(62.5)	45(51.7)	
**>147**	185	143(37.5)	42(48.3)	0.064

Abbreviation: AUC, area under the curve; CRP/Alb, C-reactive protein/albumin; GPS, Glasgow Prognostic Score; mGPS, modified GPS; NLR, neutrophil to lymphocyte ratio; and PLR: platelet lymphocyte ratio.

The optimal cutoff level for the CRP/Alb ratio was 0.50 according to the mean CRP/Alb ratio and the survival status in the 12-, 24-, and 36-month follow-up examinations. Sensitivity and specificity were 74.0% and 86.5%, respectively. We divided patients into two groups according to the cutoff level (< 0.50, n = 381; > 0.50, n = 87). The optimal cutoff level for the NLR was 2.40 for the OS, while the sensitivity and specificity for the NLR were 70.6% and 50.7%, respectively. However, the PLR was not a good prognostic marker for sensitivity and specificity; the PLR was not significant at values more than 50%. An NLR of >2.40 was found in 264 patients (56.4%), while only 185 patients (39.5%) had a PLR of >147.

### Inflammation-based factors and survival

The follow-up time of the majority (97.9%) of patients was more than 36 months or until the date of death. During this period, 259 (57.4%) patients died. The median follow-up period for the survivors was 49.9 (range 10.9–88.0) months.

**Fig 1:**
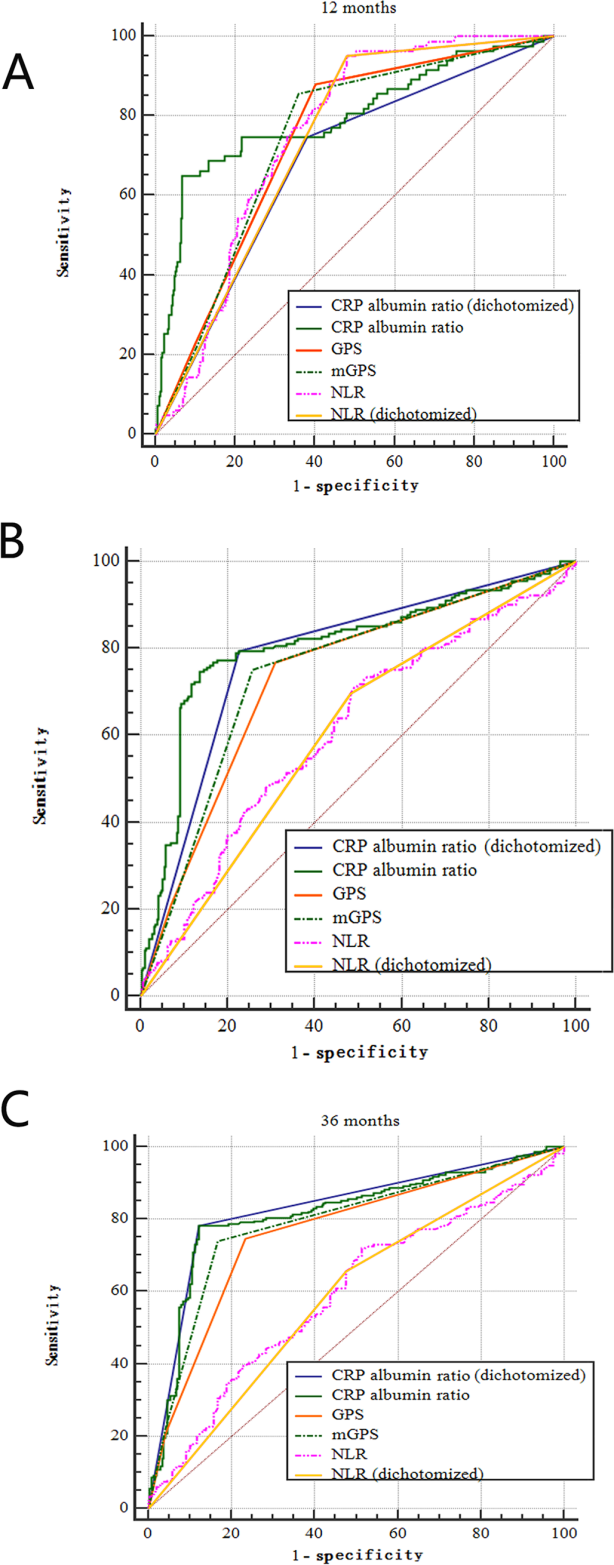
Comparison of the areas under the receiver operating curves of four inflammation—based prognostic scores [CRP/Alb ratio (continuous), GPS, mGPS, and NLR (continuous)] to prediction of overall survival at (A) 6 months, (B) 12 months, and (C) 24 months. Abbreviations: CRP/Alb, C—reactive protein/albumin; GPS, Glasgow Prognostic Score; mGPS, modified GPS; and NLR, neutrophil lymphocyte ratio.

AUC values were used to compare the ability to discriminate between the CRP/Alb ratio and the other inflammation-based prognostic scores, GPS, mGPS and NLR **(Table 2; Fig 1)**. The CRP/Alb ratio (continuous) had an AUC value at 12 months (AUC = 0.80) that was comparable to the AUC value of the NLR (continuous) (AUC = 0.75; *P* = 0.278) and higher AUC values at 24 months (AUC = 0.81) and 36 months (AUC = 0.82) than those of the NLR (continuous) (24 months: *P* < 0.0001 and 36 months: *P* < 0.0001). In addition, we compared the other two prognostic score systems based on the combination of CRP concentration and serum albumin level GPS (12 months: *P* = 0.118; 24 months: *P* = 0.009; and 36 months: *P* = 0.039) and mGPS (12 months: *P* = 0.114; 24 months: *P* = 0.021; and 36 months: *P* = 0.144). Similar results were obtained when the CRP/Alb ratio (dichotomized) was compared with the GPS, mGPS and NLR (dichotomized) (**Table 2**).

**Table 2:** Comparison of the areas under the curves for the four inflammation-based prognostic factors.

Period	AUC	95% CI	*P* value*
**12-month follow-up**			
CRP/Alb ratio			
Continuous	0.80	0.64–0.72	
Dichotomized	0.68	0.64–0.73	<0.0001
GPS	0.74	0.70–0.78	<0.118
mGPS	0.74	0.70–0.78	<0.114
NLR			
Continuous	0.75	0.71–0.79	<0.278
Dichotomized	0.74	0.69–0.77	<0.095
**24-month follow—up**			
CRP/Alb ratio			
Continuous	0.81	0.77–0.84	
Dichotomized	0.79	0.75–0.82	0.090
GPS	0.74	0.69–0.78	0.009
mGPS	0.75	0.70–0.79	0.114
NLR			
Continuous	0.61	0.507–0.66	<0.0001
Dichotomized	0.61	0.506–0.65	<0.0001
**36-month follow—up**			
CRP/Alb ratio			
Continuous	0.82	0.78–0.80	
Dichotomized	0.83	0.79–0.86	0.108
GPS	0.77	0.73–0.80	0.039
mGPS	0.79	0.75–0.83	0.244
NLR			
Continuous	0.59	0.55–0.64	<0.0001
Dichotomized	0.59	0.54–0.64	<0.0001

*The significant level of the difference in the AUC when the CRP/Alb ratio (continuous) was compared with other inflammation-based prognostic factors.

Abbreviation: AUC, area under the curve; CRP/Alb, C-reactive protein/albumin; GPS, Glasgow Prognostic Score; mGPS, modified GPS; and NLR, neutrophil to lymphocyte ratio.

**Fig 2:**
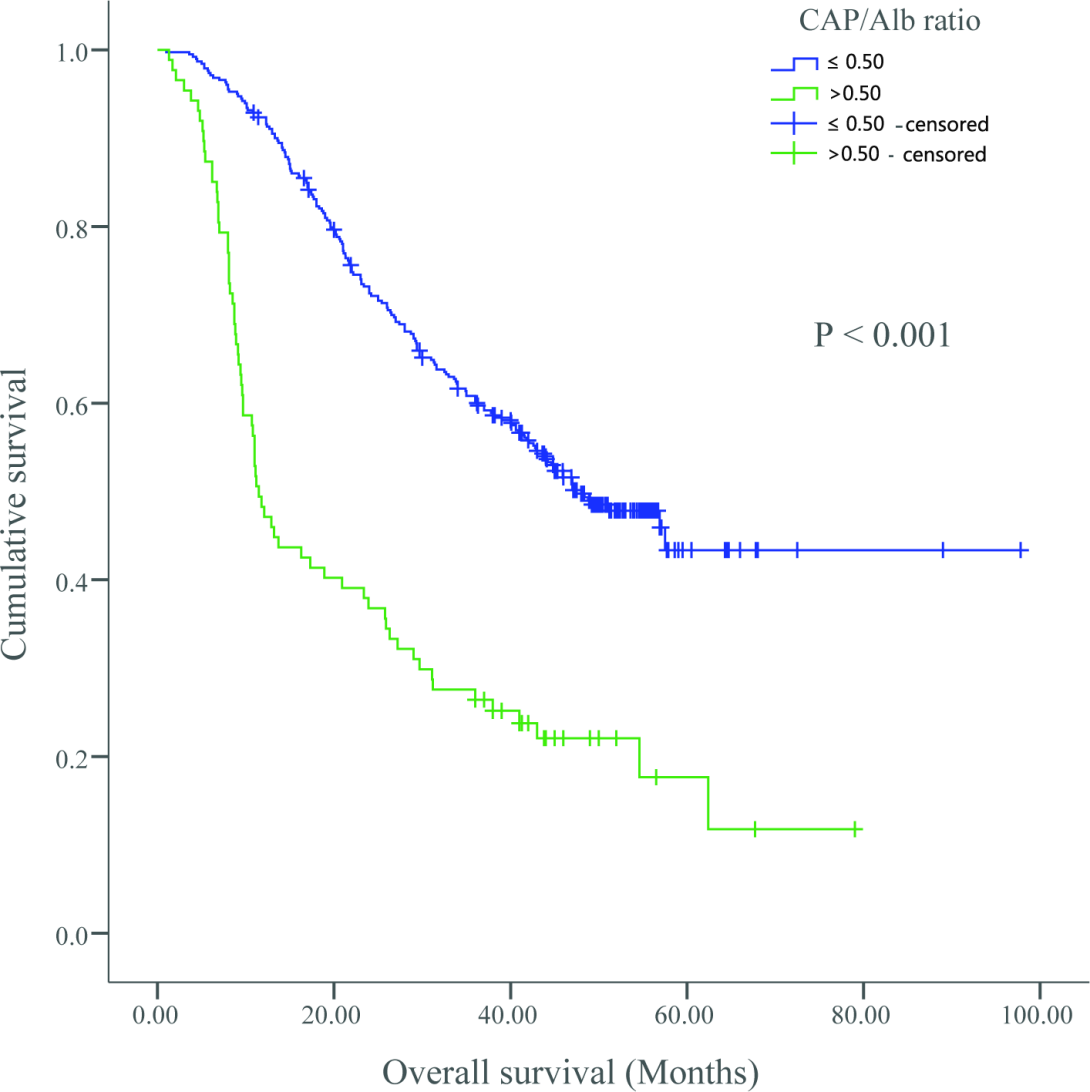
Kaplan–Meier curves showing the difference in OS for patients with primary operable ESCC categorized according to the optimal cutoff.

In univariate analysis, the 5-year OS rates for patients with a CRP/Alb ratio > 0.50 vs. patients with a CRP/Alb ratio ≤ 0.50 were 43.4% vs. 17.7% (*P* < 0.0001), respectively (**Fig 2**). Gender (*P* = 0.011), smoking (*P* = 0.033), tumor size (*P* = 0.050), T stage (*P* = 0.023), lymph node metastasis (*P* < 0.001), clinical stage (*P* < 0.0001), GPS (*P* = 0.009), mGPS (*P* = 0.007), and NLR (*P* = 0.001) were also significant predictors of OS. In multivariate analysis, patients with a CRP/Alb ratio > 0.50 had worse survival than patients with a CRP/Alb ratio ≤ 0.50 (HR: 2.44; 95% CI: 1.82–3.26; *P* < 0.0001) **(Table 3)**. In addition, lymph node metastasis (*P* < 0.0001) and venous/lymphatic invasion (*P* = 0.002) were significant independent predictors of OS (**Table 3**).

**Table 3:** Prognostic factors for overall survival identified by univariate and multivariate analyses.

Factors	Univariate analysis		Multivariate analysis	
	HR (95% CI)	*P* value of OS	HR (95% CI)	*P* value of OS
**Gender**				
**Female**	Ref.		Ref.	
**Male**	1.82(1.14–2.90)	0.011	2.72(0.93–2.47)	0.099
**Age**				
**< 58 years old**	Ref.			
**≥58 years old**	0.89(0.70–1.14)	0.352		
**Smoking**				
**Never**	Ref.			
**Ever**	1.40(1.03–1.90)	0.033		
**Alcohol consumption**				
**Never**	Ref.			
**Ever**	1.24(0.95–1.63)	0.113		
**Differentiation**				
**Well**	Ref.	0.058		
**Intermediate**	1.22(0.81–1.86)	0.344		
**Poor or undifferentiated**	1.67(1.64–1.68)	0.035		
**Tumor size**				
**<5 cm**	Ref.			
**≥5 cm**	1.30(1.00–1.70)	0.05		
**T stage**				
**T1**	Ref.	0.023		
**T2**	1.20(0.55–2.59)	0.648		
**T3**	1.82(0.90–3.68)	0.098		
**Lymph node metastasis**				
**N0**	Ref.	<0.0001	Ref.	<0.0001
**N1**	1.45(1.05–2.02)	0.027	1.44(1.02–2.02)	0.037
**N2**	2.41(1.74–3.32)	<0.0001	1.97(1.39–2.78)	<0.0001
**N3**	5.01(3.28–7.74)	<0.0001	4.21(2.66–6.65)	<0.0001
**Clinical stage**				
**I**	Ref.	<0.0001		
**II**	1.39(0.67–2.88)	0.381		
**IIIA**	2.34(1.13–4.87)	0.023		
**IIIB+IIIC**	3.92(1.91–8.05)	<0.0001		
**Venous/lymphatic invasion**				
**No**	Ref.		Ref.	
**Yes**	1.61(1.23–2.10)	<0.0001	1.50(1.17–1.94)	0.002
**Perineural invasion**				
**No**	Ref.			
**Yes**	1.63(1.27–2.10)	<0.0001		
**Adjuvant radiotherapy or chemotherapy**				
**No**	Ref.			
**Yes**	1.13(0.88–1.45)	0.33		
**CRP/Alb ratio**				
**≤0.50**	Ref.		Ref.	
**>0.50**	3.09(2.41–3.96)	<0.0001	2.44(1.82–3.26)	<0.0001
**GPS**				
**0**	Ref.	0.009		
**1**	1.33(0.99–1.78)	0.057		
**2**	1.83(1.18–2.86)	0.008		
**mGPS**				
**0**	Ref.	0.007		
**1**	1.39(1.01–1.91)	0.046		
**2**	1.82(1.17–2.83)	0.008		
**NLR**				
**≤2.40**	Ref.			
**>2.40**	1.50(1.17–1.93)	0.001		
**PLR**				
**≤147**	Ref.			
**>147**	1.12(0.87–1.43)	0.39		

Abbreviation: AUC, area under the curve; CRP/Alb, C-reactive protein/albumin; GPS, Glasgow Prognostic Score; mGPS, modified GPS; NLR, neutrophil to lymphocyte ratio; and PLR: platelet lymphocyte ratio.

A subgroup analysis of low stage patients (stage I and II) was conducted. The patients with an elevated CRP/Alb ratio still had worse survival than patients with a non-elevated CRP/Alb ratio (HR: 4.87; 95% CI: 3.11–7.63).

## Discussion

In recent years, accumulating evidence has clarified the role of inflammation-based prognostic scores [9, 12–14, 17, 21, 24–26], including the GPS, mGPS, NLR and PLR, in cancer patient prognosis. Increased levels of inflammatory cytokines such as CRP represent an inflammatory response secondary to tissue damage induced by infection, trauma, and tumor necrosis [27]. There is strong evidence that elevated CRP levels have an impact on the growth and progression of cancers, including EC [27–29]. The GPS was first introduced by Forrest *et al*. [18] who investigated its prognostic value in advanced cancer patients. McMillan *et al*. [30] proposed adopting the mGPS, which seems to be a more sensitive prognostic predictor in various malignancies. It has been reported that the GPS is associated with tumor size, depth of invasion, nodal metastasis and patient prognosis in ESCC [31, 32]. However, the role of the mGPS in ESCC patients has yet to be evaluated well.

In the present study, we calculated and compared the prognostic value of the preoperative GPS, mGPS, NLR, and CRP/Alb ratio in patients with ESCC who were treated with esophagectomy. Recently, Kinoshita *et al*. [15] showed that the CRP/Alb ratio can serve as a novel inflammation-based prognostic score to predict survival in hepatocellular carcinoma. Our study has validated the GPS, mGPS and NLR as prognostic predictors in operable ESCC patients. Interestingly, it also demonstrated that the CRP/Alb ratio may be a more sensitive prognostic predictor in patients with malignancy when the CRP/Alb ratio is defined by a cutoff level of 0.50 in survival analysis. In ROC analysis, our findings indicated that the CRP/Alb ratio may be superior to other inflammation-based prognostic scores in terms of its prognostic ability in patients with ESCC. Our results are in accordance with the results of a recently published study [33].

However, the recent study found a different "optimal" prognostic cutoff for CRP/Alb in ESCC that was significantly lower (0.095) than that in our study. This may be due to the different methods we used to determine cutoff value and the different population we enrolled. For instance, in our study we only included ESCC patients who had undergone tumor resection.

ESCC patients often suffer from cancer cachexia because they have difficulty eating, which leads to dystrophy. Complications such as palirrhea and diarrhea occurring after operation may further aggravate dystrophy. Thus, the CRP/Alb ratio, which is based on CRP and albumin, is particularly suitable for ESCC patients who have undergone esophagectomy. These blood-based biomarkers have shown promise and could reduce the worldwide health burden of ESCC by predicting prognosis. The CRP/Alb ratio (along with the GPS, mGPS and NLR) is a useful, simple, objective, reproducible, and economically feasible prognostic indicator in patients with ESCC that can be used in routine clinical laboratory tests. However, it is important to note that there are several limitations to this study. A potential limitation is that it is a retrospective, single-center study. These results need to be replicated in multicenter and prospective studies. Another limitation is that the biological mechanisms underlying the prognostic roles for systemic inflammation factors are yet to be elucidated. In addition, there is heterogeneity in the treatments for ESCC patients after surgery in our study. Finally, our cutoff value of CRP/Alb ratio is likely biased because it was selected using ROC analysis. Therefore, these results must be validated independently.

## Conclusions

In summary, the CRP/Alb ratio is a novel and promising inflammation-based prognostic factor in ESCC patients. However, the prognostic value of the CRP/Alb ratio needs to be verified in prospective multicenter studies. Further studies are warranted to validate the associations between the ratio and the prognosis and to investigate the underlying mechanisms.
